# KNDy neurons as an indirect target of insulin‐like growth factor‐1

**DOI:** 10.1111/jne.70130

**Published:** 2025-12-21

**Authors:** Josiane do N. Silva, Ligia M. M. de Sousa, Maria E. de Sousa, Henrique R. Vieira, Guilherme A. Alves, Nicole T. Neifert, Aleisha M. Moore, Jose Donato, Renata Frazao

**Affiliations:** ^1^ Department of Anatomy Instituto de Ciencias Biomedicas, Universidade de Sao Paulo Sao Paulo Brazil; ^2^ Department of Physiology and Biophysics Instituto de Ciencias Biomedicas, Universidade de Sao Paulo Sao Paulo Brazil; ^3^ Department of Biological Sciences Brain Health Research Institute, Kent State University Kent Ohio USA

**Keywords:** calcium imaging, hypothalamic–pituitary‐gonadal axis, Kisspeptin, luteinizing hormone

## Abstract

Neurons in the arcuate nucleus of the hypothalamus (ARH) that coexpress kisspeptin, neurokinin B, and dynorphin (KNDy neurons) are considered the gonadotropin‐releasing hormone (GnRH) pulse generator necessary for fertility. KNDy neurons are also metabolic sensors controlling the hypothalamic–pituitary‐gonadal (HPG) axis. Insulin‐like growth factor‐1 (IGF‐1) secretion is influenced by nutritional status and may serve as a cue detected by neurons to regulate various physiological processes, including reproduction. However, whether IGF‐1 modulates KNDy neuron activity remains unclear. RNAscope was used to assess the number of kisspeptin neurons expressing the IGF‐1 receptor (IGF1R). Additionally, the effects of IGF‐1 on LH secretion, *Kiss1* mRNA levels, intracellular calcium concentration ([Ca^2+^]i) in KNDy neurons, and resting membrane potential of kisspeptin neurons were investigated. Kisspeptin cells located at the ARH and anteroventral periventricular and rostral periventricular nuclei (here designated as AVPV) expressed the *Igf1r* in male and female mice. Intracerebroventricular IGF‐1 administration acutely increased LH secretion without altering hypothalamic *Kiss1* mRNA in male mice. In brain slices, IGF‐1 administration elevated [Ca^2+^]i in KNDy cells of male mice and depolarized KNDy neurons in both sexes. IGF‐1‐induced depolarization was abolished by TTX and amino acid receptor antagonists, indicating an indirect mechanism. In contrast, IGF‐1 has no effect on the RMP of AVPV kisspeptin neurons in female mice. IGF‐1 acutely stimulates KNDy neuron activity via indirect effects despite *Igf1r* expression in these cells. These findings identify IGF‐1 as a metabolic signal that modulates KNDy neuron excitability and, consequently, influences the reproductive axis.

## 1 INTRODUCTION

The insulin‐like growth factor 1 (IGF‐1) is a pleiotropic hormone typically produced by the liver in response to growth hormone (GH) stimulation.^1^, ^2^ GH and IGF‐1 promote body growth and development via action on organs such as the liver, skeletal muscle, and bone.^2^, ^3^ To act on the central nervous system (CNS), IGF‐1 crosses the blood–brain barrier and promotes neuronal development and cellular plasticity.^4^, ^5^ IGF‐1 also has paracrine effects within the CNS, as some neurons can produce IGF‐1, particularly during the perinatal period.^6^, ^7^ Evidence indicates that IGF‐1 and the IGF‐1 receptor (IGF1R) are essential for reproductive functions. For instance, the *Igf1* null mice are infertile.^8^ Both the gonads and the hypothalamus, tissues involved in reproduction, express IGF1R.^5^, ^9^ IGF‐1 levels increase during puberty in different species.^10^, ^11^, ^12^, ^13^, ^14^ The GH‐IGF‐1 system is crucial for folliculogenesis in the ovaries and for the proliferation of Sertoli cells and testosterone production in the testes.^1^, ^9^ IGF‐1 also contributes to the reproductive process by promoting puberty onset and stimulating the gonadotropin‐releasing hormone (GnRH) release from the hypothalamus.^12^, ^15^, ^16^ Chronic exposure to IGF‐1 is sufficient to advance puberty onset in female rats.^12^, ^15^ Central administration of IGF‐1 increases the luteinizing hormone (LH) levels in prepubertal female rats, castrated adult male sheep, and female monkeys.^12^, ^16^, ^17^, ^18^ There is also evidence that IGF‐1 is involved in the nutritional modulation of reproductive status. Adverse metabolic conditions can modulate IGF‐1 and LH secretion, potentially leading to delayed puberty.^19^, ^20^, ^21^, ^22^ However, the specific hypothalamic targets of IGF‐1 are not well defined, raising the possibility that neuronal components of the hypothalamic–pituitary‐gonadal (HPG) axis might mediate IGF‐1's effects on LH secretion.

In the arcuate nucleus of the hypothalamus (ARH), there is a neuronal population named for their coexpression of kisspeptin, neurokinin B (NKB), and dynorphin,^23^, ^24^, ^25^ the KNDy neurons. KNDy neurons are considered the pulse generator of LH secretion by acting on GnRH neurons.^26^, ^27^, ^28^ KNDy neurons have further emerged as neurons that act as sensors that inform the HPG axis about the individual's nutritional status.^29^, ^30^ Their role as mediators of metabolism occurs in part because they form complex connections with other hypothalamic neurons that mediate feeding behavior and energy homeostasis, including those expressing neuropeptide Y (NPY) and agouti‐related protein (AgRP) or pro‐opiomelanocortin (POMC) in the ARH.^31^, ^32^, ^33^, ^34^, ^35^, ^36^, ^37^ There is also communication between KNDy neurons, the paraventricular nucleus of the hypothalamus (PVN), and the dorsomedial nucleus of the hypothalamus (DMH).^38^ KNDy neurons also form intricate connections with the ventral premammillary nucleus (PMv), a critical hypothalamic nucleus for leptin's permissive action to puberty onset.^39^, ^40^ Numerous studies have demonstrated that hormones affected by changes in energy expenditure, such as leptin, insulin, ghrelin, prolactin, and GH, impact KNDy neuron activity.^30^, ^32^, ^41^, ^42^, ^43^, ^44^, ^45^, ^46^, ^47^ Accordingly, changes in energy homeostasis influence KNDy neuronal activity and affect the HPG axis.^29^, ^30^, ^48^, ^49^, ^50^, ^51^, ^52^ Despite these insights, the role of IGF‐1 in modulating KNDy neuron function remains unclear.

We hypothesize that IGF‐1 influences KNDy neuron activity and, consequently, the HPG axis. We used gonad‐intact male and female mice to explore the potential effects of IGF‐1 on kisspeptin neurons. We employed several experimental approaches, such as RNAscope in situ hybridization, central administration of IGF‐1, *Kiss1* mRNA expression, calcium imaging, and whole‐cell patch‐clamp analysis, to determine whether IGF‐1 affects the activity of kisspeptin neurons.

## 2 MATERIALS AND METHODS

### 2.1 Animals

We performed experiments using male and female C57Bl/6J and Kiss1‐hrGFP mice (8–12 weeks of age, Stock No: 023425, The Jackson Laboratory, Bar Harbor, ME). Mice were weaned at 3 weeks of age, and Kiss1‐hrGFP mice were genotyped via PCR to confirm the presence of the hrGFP expression under the transcriptional control of the *Kiss1* gene.^44^ The DNA was extracted from tail tips using the REDExtract‐N‐Amp Tissue PCR Kit (Sigma, St. Louis, MO). Mice were kept in cages with four to five animals per group.

Animals were kept in environmentally controlled rooms with a 12 h on/12 h off light cycle (lights on at 06:00 am) and a temperature of 21–23°C. The Institute of Biomedical Sciences Animals Ethics Committee at the University of São Paulo and the Kent State University Institutional Animal Care and Use Committee approved all animal procedures.

### 2.2 RNAscope

C57BL/6J female mice in diestrus (*n* = 4), as determined by vaginal cytology, and male mice (*n* = 3/4) were perfused transcardially between 10:00 and 12:00 with 4% paraformaldehyde in 0.1 M phosphate‐buffered saline (PBS). Brains were extracted and incubated in the same fixative overnight at 4°C and then sunk in 10%, 20%, and 30% sucrose in 0.1 M PBS. Brains were rapidly frozen in optimal cutting temperature (OCT) compound (FisherScientific) and cryosectioned into 12 μm‐thick coronal sections on super‐frost charged slides. To assess the co‐expression of *Kiss1* mRNA and *Igf1r* mRNA in the ARH, or in the anteroventral periventricular and rostral periventricular nuclei (here designated as AVPV), in situ hybridization was performed with the RNAscope Multiplex Fluorescent Reagent V2 Kit (ACD Biotechne) using instructions provided by the manufacturer. Negative (Cat # 320871) and positive (Cat # 320881) control probes and probes against *Kiss1* (Cat # 500141) and *Igf1r* (Cat # 417561) were used. Probe binding was visualized with Cy3 and Cy5 fluorophores, and cell nuclei were identified with Dapi labeling. Two sections containing the middle ARH (−2.06 mm posterior to Bregma) and two sections containing the AVPV (0.2 mm anterior to Bregma) were imaged per animal using an Olympus FV3000 confocal microscope with a 40× objective, and optical sections with a 2 μm step size were collected. The middle ARH sections were chosen for RNAscope analysis, as this subsection of kisspeptin cells has a demonstrated role in GnRH pulse generation.^26^, ^53^ In the ImageJ software, the number of DAPI cells containing fluorescent mRNA transcripts for *Kiss1* alone or *Kiss1* and *Igf1r* was counted, and the percentage of kisspeptin cells containing *Igf1r* was calculated and reported as the average per animal. In addition, the number of *Igf1r* mRNA transcripts, as identified via fluorescent puncta, was quantified and averaged per section and then per animal. ARH and AVPV sections labeled with the negative control probe were used to determine non‐specific fluorescence, and a cell was deemed to express a target gene when three or more transcripts overlaid DAPI.

### 2.3 Effects of central IGF‐1 injection on LH serum levels

Adult male Kiss1‐hrGFP mice were daily adapted to tail‐tip blood sampling for 30 days to minimize stress on the experimental day. Four days before the blood collection and perfusion, mice were implanted with a cannula (P1 Technologies; Roanoke, Canada) in the lateral ventricle.^54^ On an experimental day, mice received a 2 μL intracerebroventricular (icv) injection of saline (*n* = 7) or mouse recombinant IGF‐1 (0.5 μg/μL; *n* = 9). Ten minutes after the icv injection, a 5 μL blood sample was collected from the tail tip, transferred to 105 μL of phosphate‐buffered saline (PBS) containing 0.05% Tween‐20 (PBS‐T), placed on dry ice, and stored at −80°C. As previously described, LH concentrations were assessed using an in‐house enzyme‐linked immunosorbent assay,^55^ with a lower limit of detection of 0.129 ng/mL and intra‐ and inter‐assay coefficients of variation of 4.43% and 7.51%, respectively.

### 2.4 Effects of central IGF‐1 injection on Kiss1 mRNA expression

A subgroup of mice was sacrificed 5–6 h (3:00–4:00 pm) after the icv administration of saline (*n* = 5) or mouse recombinant IGF‐1 (0.5 μg/μL; *n* = 8), to determine its potential effects on *Kiss1* gene expression in the ARH or the AVPV. Brain slices and micro‐punches were performed based on anatomical references according to the atlas (Allen Brain Institute), as previously described.^56^ A single midline punch of the AVPV (bregma 0.38 to 0.02 mm) was obtained from a coronal brain section of 250 μm. Bilateral punches of the ARH were obtained from a coronal brain slice of 999 μm (relative to bregma −1.46 to −2.46 mm) using an 18‐gauge needle. Total RNA from the micro punches was extracted using the PicoPure RNA isolation kit (Thermo Fisher Scientific). An Epoch Microplate Spectrophotometer (Gen5, BioTek; RRID: SCR_017317) was used to determine RNA quantity. Reverse transcription was performed by combining 0.25 μg of total RNA from the AVPV, or 0.25 μg of total RNA from ARH, the SuperScript II Reverse Transcriptase (Thermo Fisher Scientific), and random primers p(dN)6 (Roche Applied Science). Real‐time polymerase chain reaction (RT‐PCR) was performed using a 7500 Real‐Time PCR System (Thermo Fisher Scientific) and the Power SYBR Green PCR Master Mix (Thermo Fisher Scientific). All samples were run in duplicate, and negative controls were included on each qPCR plate. The *Kiss1* relative mRNA expression was quantified by calculating 2^−ΔΔCt^. Data were normalized to the geometric average of the housekeeping genes *Actb* and *Ppia* and reported as fold change compared to values obtained from the control group (set to 1.0). The genes and primer set for the RT‐PCR analysis were as follows: *Actb* (forward: gctccggcatgtgcaaag; reverse: catcacaccctggtgccta), *Kiss1* (forward: gattccttttcccaggcatt; reverse: ggcaaaagtgaagcctggat), and *Ppia* (forward: cttcttgctggtcttgccattcc; reverse: tatctgcactgccaagactgagt). We observed no significant differences in the average cycle threshold (Ct) values for *Actb* or *Ppia* in the ARH or the AVPV between saline or IGF‐1‐treated mice (data not shown).

### 2.5 Calcium imaging

Male Kiss1‐hrGFP mice were anesthetized with isoflurane and euthanized by decapitation. The brains were quickly removed and immediately submerged in ice‐cold, carbogen‐saturated (95% O_2_ and 5% CO_2_) artificial cerebral spinal fluid (ACSF) composed of 238 mM sucrose, 2.5 mM KCl, 26 mM NaHCO_3_, 1 mM NaH2PO_4_, 5 mM MgCl_2_, 10 mM d‐glucose, and 1 mM CaCl_2_. Coronal brain slices were cut (200 μm) using a vibratome. Brain slices were submerged in carbogen‐saturated ACSF containing 125 mM NaCl, 3 mM KCl, 1 mM MgCl_2_, 2 mM CaCl_2_, 25 mM NaHCO_3_, and 10 mM d‐glucose, as described.^57^ After 60 min, brain slices were incubated in a ratiometric fluorescent dye that can bind to intracellular calcium (Fura‐2, AM, Thermo Fisher Scientific). Fura‐2 was diluted (50 μg of Fura‐2 in 48 μL of DMSO and 2 μL of Pluronic F‐127), and brain slices were incubated in 1 mL of carbogen‐saturated ACSF and FURA‐2 (for a final concentration of 10 μM)^58^ for 60 min at room temperature. After incubation, brain slices were transferred to a recording chamber and washed with carbogen‐saturated ACSF (30°C, flow rate of 2 mL/min) for 30 min.

Brain slices were imaged on an inverted Leica DM6000 FS microscope with a fixed stage and a Leica DFC360 FX high‐speed monochrome fluorescence digital camera. Images were visualized through a 20× immersion objective, and epifluorescence was collected using Leica's LAS X software at a 1 Hz frame rate. ARH was assessed, covering the anteroposterior distance −1.46 to −2.70 mm rostral to bregma. The IGF‐1 effects were evaluated by measuring basal fluorescence during a baseline period (2–5 min), followed by IGF‐1 administration to the bath (0.5 μg/μl) for 5 min, and switching back to ACSF (washout period, 5–10 min). Regions of interest (ROIs) were drawn around neurons, and [Ca^2+^]i was calculated based on the fluorescence ratio of the two, 340 nm and 380 nm, excitation wavelengths.^59^ At the end of the recordings, cell viability was tested by adding L‐glutamic acid (100 μM) to the bath. Only L‐glutamic acid‐responsive cells were considered in the analyses. Average fluorescence obtained from 1 min of the baseline period (immediately before drug application) and 1 min during the IGF‐1 exposure (immediately before washout) was used for analysis, allowing for a consistent comparison of responses across cells. Individual cells were considered responsive if they exhibited changes in [Ca^2+^]i greater than two times the standard deviation from the mean [Ca^2+^]i during the baseline period.^60^, ^61^

### 2.6 Electrophysiological recordings

To determine IGF‐1 effects on the resting membrane potential (RMP) of the ARH and the AVPV kisspeptin cells, male or female (diestrus) Kiss1‐hrGFP mice (*n* = 7/9 mice per sex) were anesthetized with isoflurane, decapitated, and their brains were quickly removed and submerged in ice‐cold carbogen‐saturated ACSF, as mentioned.

Coronal brain slices were made (250 μm), and kisspeptin neurons were identified as described.^62^ The brain slices were maintained in carbogen‐saturated (95% O_2_ and 5% CO_2_) ACSF containing 135 mM NaCl, 3.5 mM KCl, 26 mM NaHCO_3_, 1.25 mM NaH_2_PO_4_, 1.2 mM MgSO_4_, 10 mM glucose, and 1 mM CaCl_2_ at 30°C for at least 1 h before the experiment. The micropipette solution was composed of 130 mM K ‐gluconate, 15 mM KCl, 10 mM HEPES, 5 mM EGTA, 0.1 mM CaCl_2_, 4.0 mM MgATP, 0.4 mM NaGTP, and 8.5 mM NaOH, pH 7.3. The membrane potential was monitored for at least 5 min (baseline), followed by administration of IGF‐1 (0.5 μg/μL) to the bath. The effects evoked by IGF‐1 were monitored for up to 10 min. Further analyses were performed as previously reported.^56^ The input resistance (IR) was assessed by measuring the voltage deflection at the end of the response to a hyperpolarizing rectangular current pulse (500 ms of −10 to −30 pA). Recordings of ARH kisspeptin neurons were also performed with ACSF containing tetrodotoxin (TTX, 1 μM), CNQX (10 μM), AP‐5 (50 μM), and picrotoxin (50 μM) to assess IGF‐1 effects on action potentials (APs) mediated by synaptic transmission (*n* = 5/6 mice per sex). The membrane potential values were compensated to account for the liquid junction potential (−15 mV). The electrophysiological signals from kisspeptin neurons were recorded using an Axopatch 700B amplifier (Molecular Devices; RRID: SCR_011323), low‐pass filtered at 2–4 kHz, and analyzed offline on a PC using the pCLAMP program (Molecular Devices). The APs frequency (*fAPs*) was determined by comparing the average firing rates 2 min immediately before and 2 min while applying the drugs to the recording chamber. The *fAPs* data were analyzed with the Mini Analysis Program (Synaptosoft, Decatur, GA; RRID: SCR_002184). For all recordings, series resistance was <20 MΩ, and data were discarded if a change of 20% or more occurred during the recording.

### 2.7 Drugs

The IGF‐1 concentration was based on previous studies demonstrating significant IGF‐1 effects on puberty onset.^63^ The concentrations of TTX and the amino acid receptor antagonists were based on previous studies showing their abilities to block Na^+^ conductance (TTX), ionotropic glutamatergic receptors (CNQX, AP‐5), and GABA_A_ receptors (picrotoxin).^56^, ^64^ The mouse IGF‐I recombinant protein was obtained from Thermo Fisher Scientific. TTX was obtained from Alomone Labs. CNQX, AP‐5, and picrotoxin were purchased from Tocris Bioscience.

### 2.8 Statistical analysis

Statistical analyses were performed using the GraphPad Prism software (GraphPad Prism; RRID: SCR_002798). The data were tabulated and presented as mean ± SE of the mean in graphs. RNAscope and RT‐PCR were compared using an unpaired two‐tailed Student's *t*‐test. The Mann–Whitney *U* test was used to evaluate [Ca^2+^]i data. Repeated‐measures ANOVA and Tukey's post‐test were used to assess RMP and IR data. Friedman and Dunn's multiple tests were used to evaluate IGF‐1 effects on the *fAPs*. Fisher's exact test was used to compare the number of responsive cells. Results with *p*‐values of <0.05 were considered statistically significant.

## 3 RESULTS

### 3.1 Kisspeptin neurons express the IGF1R

**FIGURE 1:**
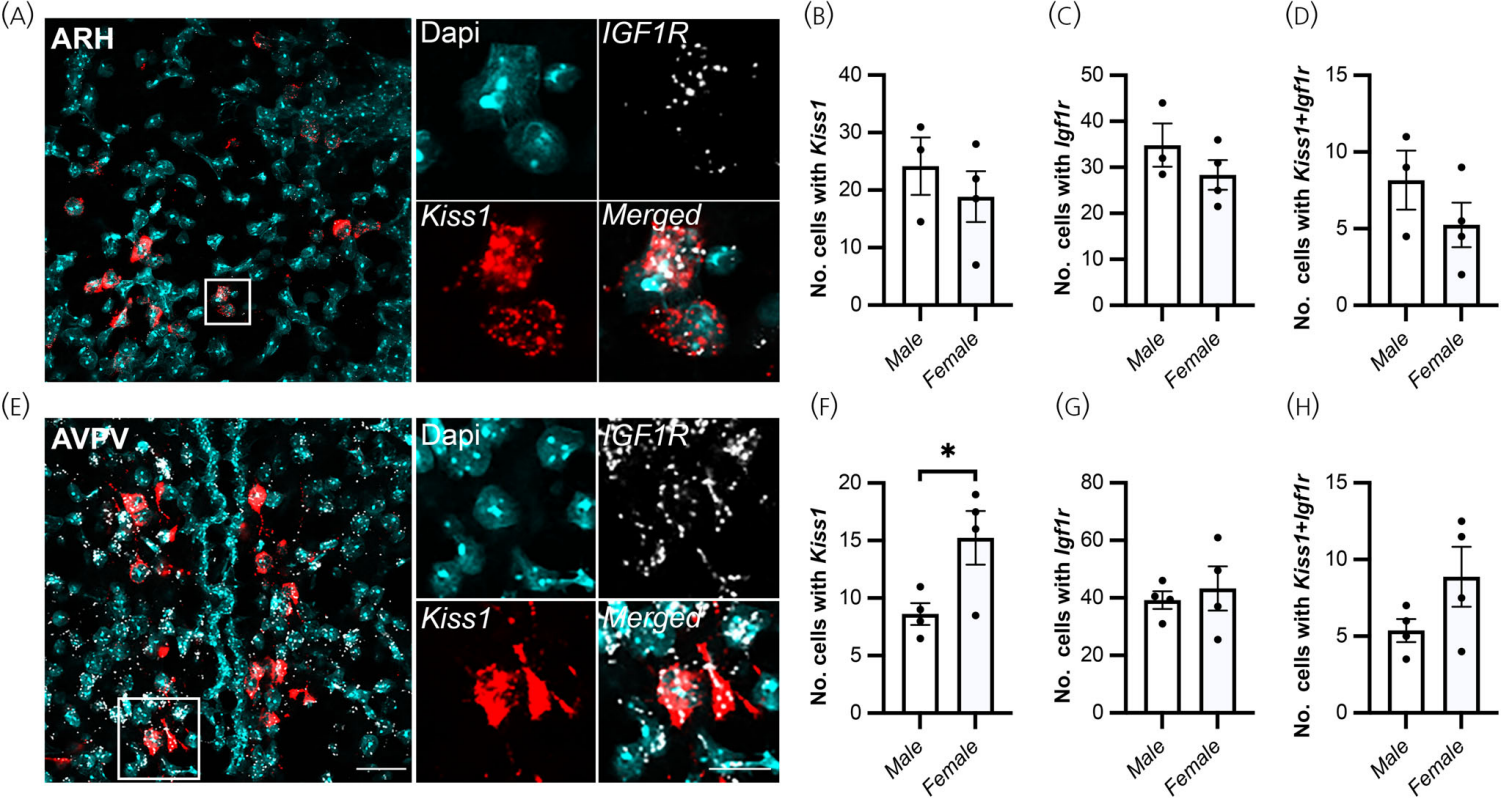
A subpopulation of ARH kisspeptin (presumptively KNDy) and AVPV kisspeptin cells expresses *Igf1r* mRNA in male and female mice. (A, E) Representative confocal image showing the multiplex detection of *Kiss1* (red) and *Igf1r* (white) mRNA overlaying Dapi‐labelled cell nuclei (cyan) in the ARH (A) and the AVPV (E). Magnified insets show examples of cells co‐expressing *Kiss1* and *Igf1r* mRNA. Bar = 25 μm. (B–D, F–H) Bar graph demonstrating the average number of cells with *Kiss1* (B, F), the average number of cells with *Igf1r* (C, G), and that a subset of KNDy (D) or AVPV (H) cells co‐express *Igf1r* in male (*n* = 3/4) and diestrus female (*n* = 4) mice, but no significant sex difference was detected (unpaired *t*‐test, *p* >0.05). Bar = 10 μm.

To investigate whether kisspeptin neurons express IGF1R, in situ hybridization was used to colocalize *Kiss1* and *Igf1r* mRNA (Figure 1A,E). The total number of *Kiss1*‐mRNA expressing cells in the ARH was similar between male (24.2 ± 5.0 cells) and diestrus female mice (18.9 ± 4.4 cells; *p* = 0.5; Figure 1A,B). In contrast, the total number of *Kiss1*‐mRNA expressing cells in AVPV was lower in male (8.6 ± 0.9 cells) compared to female mice (15.3 ± 2.3 cells; *p* = 0.03; Figure 1E,F), as previously reported.^65^, ^66^ The average number of Dapi‐labelled cells expressing fluorescent *Igf1r* mRNA puncta within ARH (male, 34.8 ± 4.7 cells; female, 28.4 ± 3.2 cells; Figure 1C) or AVPV (male, 39.3 ± 3.1 cells; female, 43.3 ± 7.7 cells; Figure 1G) cells did not significantly differ between males and females (ARH, *p* = 0.3; AVPV, *p* = 0.6). A subset of kisspeptin cells in both males and females co‐expressed *Igf1r* mRNA, with no significant difference in co‐expression observed between the sexes either in the ARH (male, 8.2 ± 1.9 out of 24.2 ± 5.0 cells; female, 5.3 ± 1.5 out of 18.9 ± 4.4 cells, *p* = 0.3; Figure 1D) or the AVPV (male, 5.4 ± 0.6 out of 8.6 ± 0.9; female, 8.9 ± 2.0 out of 15.3 ± 2.3 cells, *p* = 0.1; Figure 1H). The number of *Igf1r* puncta within kisspeptin cells, which indicates the level of mRNA expression, did not differ significantly between males and females (ARH, male, 4.3 ± 1.3; female, 3.2 ± 0.2, *p* = 0.4; AVPV, male, 3.1 ± 0.1; female, 3.0 ± 0.0, *p* = 0.4). Similarly, no differences in the percentage of *Kiss1‐*expressing *Igf1r* cells in the ARH (male, 27.7 ± 1.9%; female, 33.2 ± 3.8%, *p* = 0.2) and AVPV (male, 63.1 ± 1.2%; female, 56.9 ± 7.9%, *p* = 0.3) were calculated.

### 3.2 IGF‐1 induces LH secretion in male mice

**FIGURE 2:**
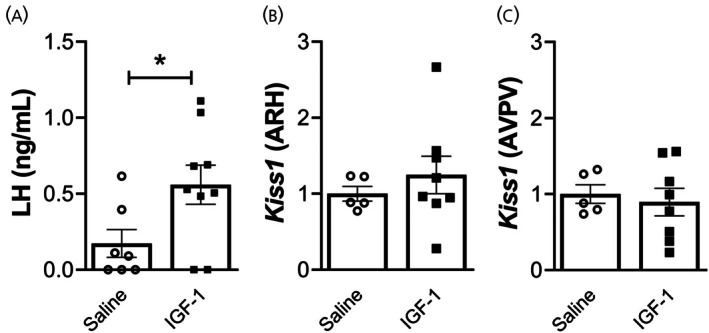
IGF‐1 stimulates LH secretion despite no evident effect on *Kiss1* mRNA levels. (A) Bar graphs demonstrating the effects of intracerebroventricular (icv) administration of saline (*n* = 7) or IGF‐1 (0.5 μg/μL, *n* = 9) on luteinizing hormone (LH) secretion 10 min after icv administration. (B, C) Bar graphs demonstrating the effects of icv administration of saline (*n* = 5) or IGF‐1 (0.5 μg/μL, *n* = 8) on *Kiss1* mRNA levels at the ARH (B) and the AVPV (C) 5–6 h after icv administration (unpaired *t*‐test, *p < 0.05).

Previous studies have suggested that IGF‐1 can induce LH secretion in various species.^12^, ^15^, ^16^, ^18^ However, the capacity of IGF‐1 to affect LH secretion has never been explored in gonad‐intact male mice. To determine whether IGF‐1 is sufficient to induce LH secretion in male mice, animals received an icv injection of saline (*n* = 7) or IGF‐1 (*n* = 9), and blood LH levels were determined 10 min later. IGF‐1‐treated male mice exhibited increased LH serum levels compared to saline‐injected animals (Figure 2A, *p* = 0.03).

### 3.3 IGF‐1 does not modulate hypothalamic *Kiss1* mRNA levels

We next investigated whether icv IGF‐1 administration influences *Kiss1* mRNA expression. After 5–6 h of saline (*n* = 5) or IGF‐1 (*n* = 8) icv administration, we collected micropunches from ARH and AVPV. Our analysis revealed no significant changes in *Kiss1* mRNA levels in the ARH (*p* = 0.5) or AVPV (*p* = 0.7) between IGF‐1 and saline‐treated mice (Figure 2B,C). These findings indicate that icv administration of IGF‐1 does not modulate hypothalamic *Kiss1* mRNA levels in adult male mice, at least within the chosen time frame.

### 3.4 IGF‐1 leads to increased intracellular calcium levels in ARH kisspeptin neurons

**FIGURE 3:**
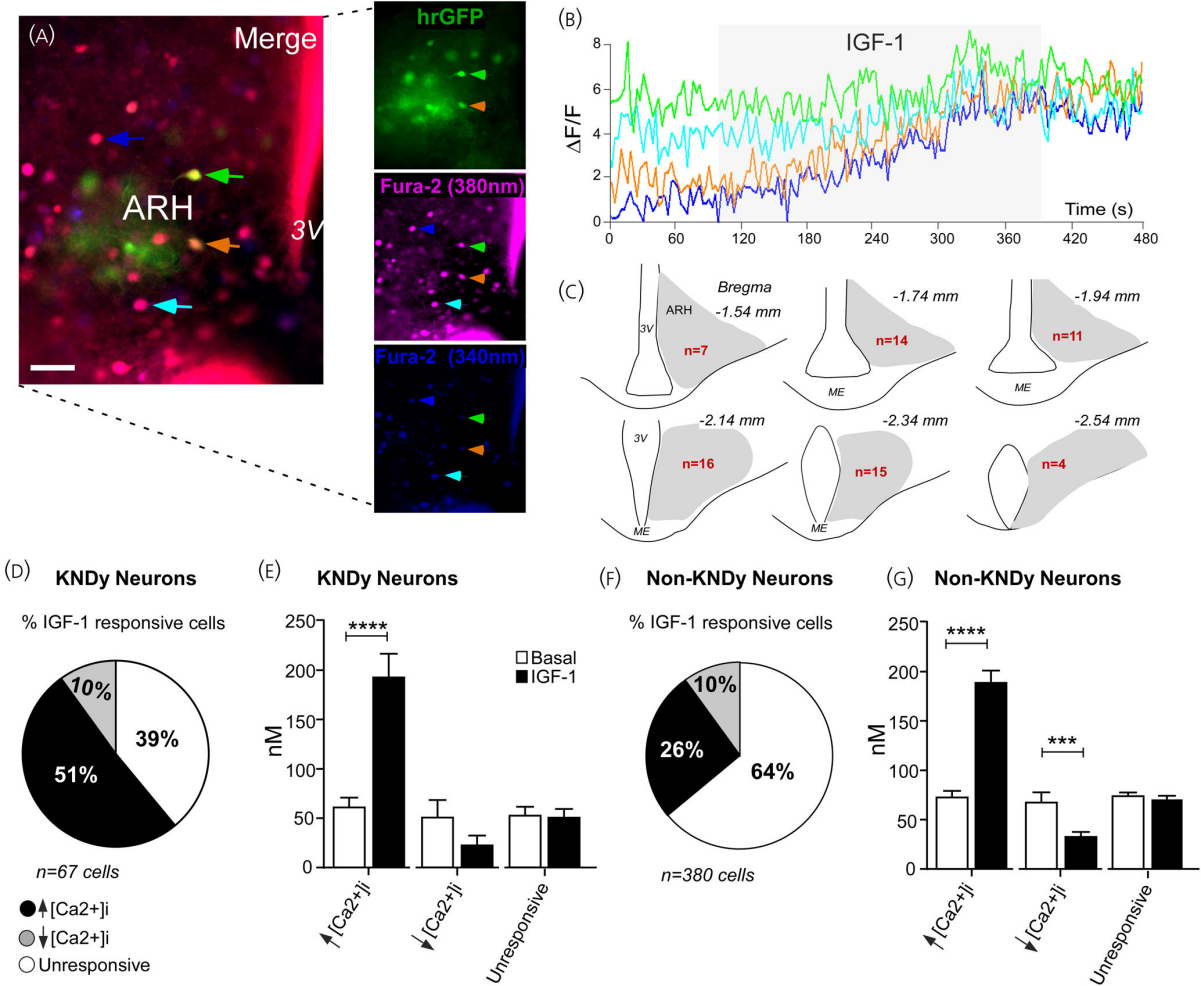
IGF‐1 triggers an elevation in the intracellular calcium levels in most ARH kisspeptin (presumptively KNDy) neurons. (A) Epifluorescence photomicrographs showing the distribution of KNDy (green) and non‐KNDy cells, which incorporated FURA‐2 (purple, light spectrum emitted at 380 nm indicating calcium‐free Fura‐2; blue, light spectrum emitted at 340 nm indicating Fura‐2 in the calcium‐bound state). Green and orange arrows indicate KNDy neurons incorporating Fura‐2; light blue and dark blue refer to non‐KNDy neurons incorporating Fura‐2. B. Representative tracings refer to the cells indicated by the colorful arrows in “A.” (C) Schematic drawing representing the rostrocaudal levels of the arcuate nucleus of the hypothalamus (ARH), where KNDy neurons responsive to IGF‐1 were recorded. (D, F) The percentage of KNDy (D) and non‐KNDy neurons (F) in which IGF‐1 modulates the concentration of the intracellular calcium levels ([Ca^2+^]i). (E, G) Average [Ca^2+^]i of KNDy (E) and non‐KNDy neurons (G) recorded in the ARH. 3 V, third ventricle; hrGFP, human renilla green fluorescent protein. Bar = 30 μm; *N* = 447 cells recorded from 26 male mice (Mann–Whitney *U* test, ****p = 0.0001).

To assess whether IGF‐1 modulates intracellular Ca^2+^ levels of ARH kisspeptin neurons in male mice, we performed calcium imaging analyses using Fura‐2 as a Ca^2+^ indicator. Fura‐2 was incorporated by ARH kisspeptin and non‐kisspeptin neurons located in the ARH (Figure 3A). Out of 447 cells that incorporated FURA‐2, 67 cells were recognized as ARH kisspeptin neurons.^44^ ARH kisspeptin neurons exhibited a lower mean [Ca^2+^]i (57.8 ± 5.6 nM; *n* = 67 cells) compared to non‐kisspeptin neurons (73.1 ± 2.9 nM; 380 cells; *p* = 0.04).

IGF‐1 administration to the bath led to a significant increase in mean [Ca^2+^]i in 51% of ARH kisspeptin neurons evaluated (34 out of 67 cells; IGF‐1, 192.8 ± 23.9 nM; baseline period, 61.7 ± 9.1 nM; *p* < 0.0001; Figure 3B–E). Non‐responsive cells (39%, 26 of 67 neurons) exhibited an average [Ca^2+^]i of 51.9 ± 6.5 nM after IGF‐1 application versus 54.2 ± 7.1 nM in the baseline period (*p* = 0.8; Figure 3D,E). Among the ARH kisspeptin cells recorded, seven out of the 67 neurons (10%) exhibited a decrease in mean [Ca^2+^]i during IGF‐1 administration to the bath (24.4 ± 8.1 nM) compared to the baseline period (52.2 ± 16.4 nM). However, the decrease of [Ca^2+^]i in this subset of cells failed to reach significance (*p* = 0.2). During baseline recordings, ARH kisspeptin‐responsive and non‐responsive neurons exhibited similar mean [Ca^2+^]i (*p* = 0.7; Figure 3E).

In contrast, most of the non‐kisspeptin neurons (64%, 242 out of 380 cells) were not responsive to IGF‐1 administration to the bath (IGF‐1, 70.2 ± 3.8 nM, versus baseline period, 73.9 ± 3.7 nM; *p* = 0.2; Figure 3F,G). IGF‐1 also modulated [Ca^2+^]i in a subgroup of non‐kisspeptin neurons. Approximately 26% of non‐kisspeptin neurons (100 out of 380 cells) exhibited increased [Ca^2+^]i due to IGF‐1 administration to the bath (IGF‐1, 188.6 ± 12.5 nM, versus baseline period, 73.1 ± 6.0 nM, *p* < 0.0001; Figure 3F,G). In addition, IGF‐1 decreased mean [Ca^2+^]i in 10% of non‐kisspeptin neurons recorded (38 of 380 cells; IGF‐1, 32.8 ± 4.6 nM, versus baseline period, 67.8 ± 9.7 nM, *p* = 0.0001).

Therefore, IGF‐1 modulated [Ca^2+^]i in most of the ARH kisspeptin cells. This effect was not exclusive to this neuronal population.

### 3.5 IGF‐1 depolarizes ARH kisspeptin neurons

**FIGURE 4:**
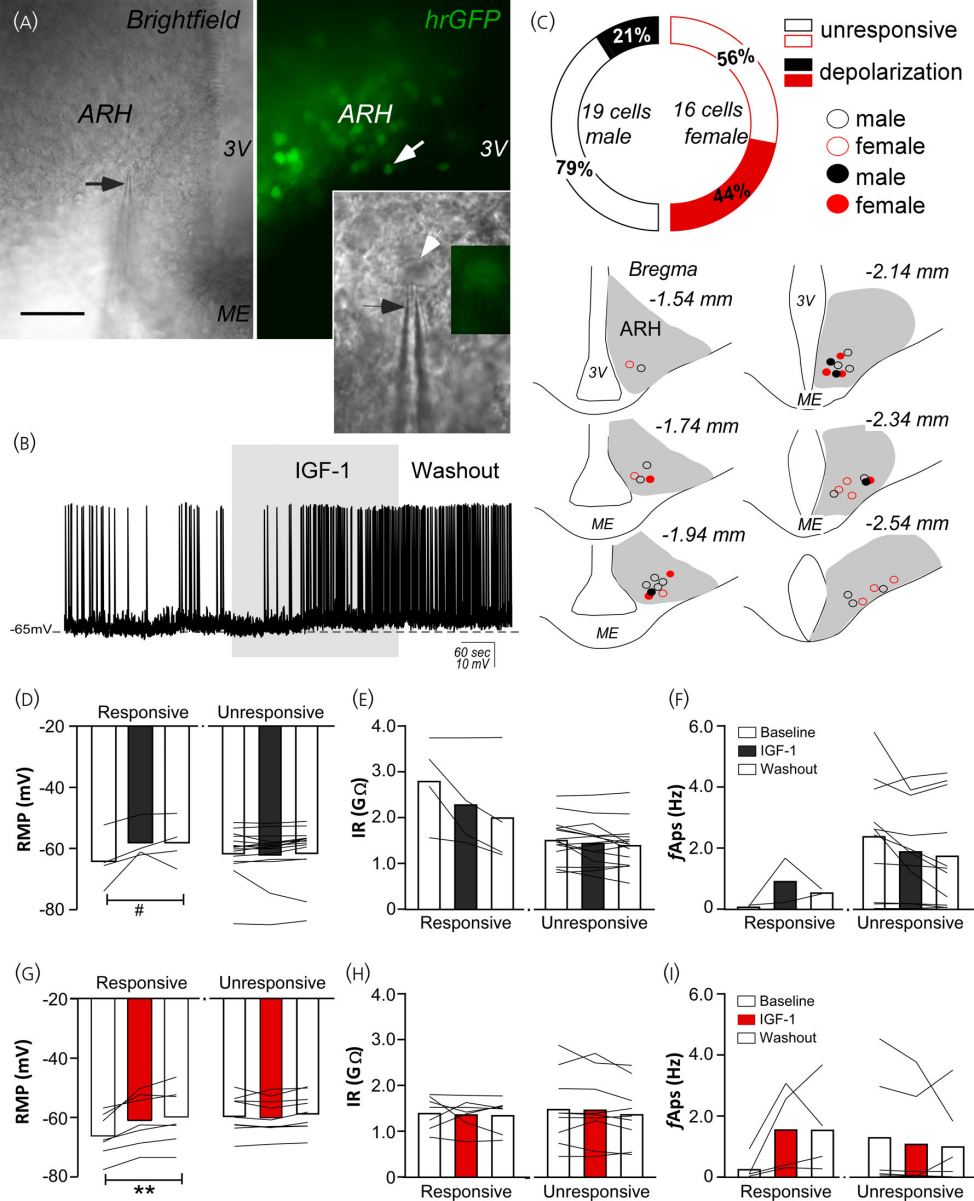
IGF‐1 depolarizes ARH kisspeptin (presumptively KNDy) neurons. (A) Representative photomicrographs of the arcuate nucleus of the hypothalamus (ARH) containing KNDy neurons (green, the black arrow points to the recording pipette, and the white arrow points to the recorded cell). (B) Representative current‐clamp recording demonstrating that IGF‐1 can depolarize KNDy neurons' resting membrane potential (RMP). (C) A representative graph showing the percentage of KNDy cells responsive to IGF‐1 in male and female mice and a schematic drawing representing the number of KNDy cells recorded throughout the rostrocaudal levels of the ARH. (D–I) Bar graphs demonstrating the IGF‐1 effects on average RMP, input resistance (IR), and the frequency of spontaneous action potential (*f*Aps) from cells recorded from male (D–F) and female mice (G–I). 3 V, third ventricle; ME, median eminence; hrGFP, human renilla green fluorescent protein. Bar = 50 μm. *N* = 8/9 mice per sex (paired one‐way ANOVA, followed by Tukey's test, ^#^ *p* = 0.05, **p = 0.002).

Next, we performed whole‐cell patch‐clamp recordings to determine whether the observed effects on [Ca^2+^]i were sufficient to alter the RMP of ARH kisspeptin neurons. Brain slices obtained from male mice were used to determine IGF‐1 effects (0.5 μg/μL) on the RMP of ARH kisspeptin cells. The electrophysiological recordings were performed in current‐clamp mode (I = 0). ARH kisspeptin neurons recorded from male mice exhibited an RMP of −61.7 ± 1.8 mV and IR of 1.8 ± 0.2 GΩ at the baseline period (19 cells from 8 animals). Thirteen out of 19 cells exhibited APs at rest (2.3 ± 0.5 Hz). The administration of IGF‐1 led to depolarization in four out of 19 cells recorded (≈21%; Figure 4B–D; *p* = 0.05). IGF‐1‐mediated depolarization was followed by a slight, although not significant, decrease in IR (*p* = 0.1) and an increase in the *fAPs* (2 out of 4 responsive cells exhibited APs at rest, Figure 4E,F; *p* = 0.5). Most cells recorded from male mice (≈82%) were unresponsive to IGF‐1 administration to the bath (Figure 4C–F; *p* > 0.05).

Next, we investigated whether similar effects could be observed when IGF‐1 was evaluated in brain slices from female mice (diestrus). ARH kisspeptin neurons of female mice exhibited similar RMP (−62.1 ± 1.8 mV; *p* = 0.9), IR (1.4 ± 0.1 GΩ, 16 cells from 9 animals, *p* = 0.1), and *fAPs* at rest (10 out of 16 cells exhibited APs at rest, 1.1 ± 0.5 Hz; *p* = 0.2) compared to male data, as demonstrated previously.^30^, ^64^ In female mice, IGF‐1 depolarized seven out of 16 recorded neurons (≈44% of the recorded cells; Figure 4C,G; *p* = 0.002). IGF‐1 effects on RMP were not followed by changes in IR (Figure 4H; *p* = 0.8). Among cells responsive to IGF‐1 and exhibiting APs at rest (4 out of 7 cells), we observed a slight increase in the *fAPs* (Figure 4I; *p* = 0.2). The remaining recorded cells from female mice (≈56%) were unresponsive to IGF‐1 (Figure 4G–I). The number of ARH kisspeptin IGF‐1‐responsive cells was similar between male and female mice (*p* = 0.3).

We further evaluated the potential effects of IGF‐1 on the RMP of AVPV kisspeptin neurons. AVPV kisspeptin neurons recorded from female mice exhibited an RMP of −76.9 ± 2.3 mV and an IR of 0.5 ± 0.1 GΩ at resting (11 cells from 7 animals). In contrast to ARH, IGF‐1 exerted no effect on the RMP of AVPV kisspeptin neurons (IGF‐1, −76.3 ± 2.6 mV, *p* = 0.8) or IR (IGF‐1, 0.4 ± 0.1 GΩ, *p* = 0.1).

### 3.6 IGF‐1 exerts indirect effects on ARH kisspeptin neurons

**FIGURE 5:**
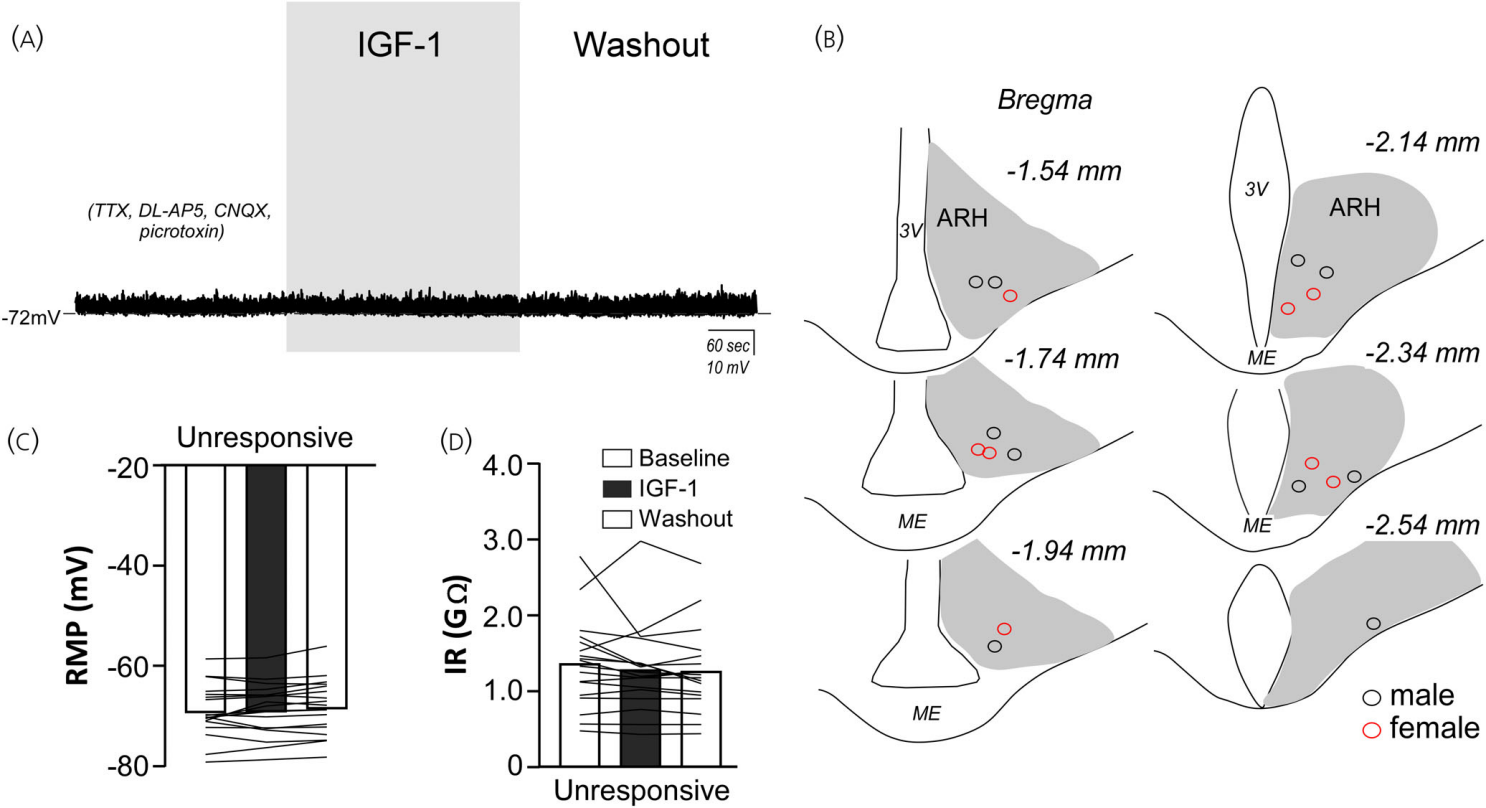
IGF‐1 effects on ARH kisspeptin (presumptively KNDy) neurons are indirect. (A) Representative current‐clamp recording demonstrating that IGF‐1 exert no effect on KNDy neurons' resting membrane potential (RMP) when recordings were performed in ACSF containing tetrodotoxin (TTX, 1 μm) and the amino acid receptor antagonists (6‐cyano‐7‐nitroquinoxaline‐2,3‐dione (CNQX, 10 μm), 2‐amino‐5‐phosphonovalerate (AP‐5, 50 μm) and picrotoxin (50 μm). (B) Schematic drawing representing the number of recorded KNDy cells in the rostrocaudal levels of the arcuate nucleus of the hypothalamus (ARH) in male and female mice. (C) Bar graphs demonstrate the effects of IGF‐1 on average RMP and input resistance (IR) when recordings are performed in ACSF containing TTX and the amino acid receptor antagonists (male and female data are shown together). 3 V, third ventricle; ME, median eminence. *N* = 5/6 mice per sex (paired one‐way ANOVA, followed by Tukey's test, *p* > 0.05).

To determine whether IGF‐1 effects on the RMP of ARH kisspeptin neurons were direct on the recorded cells, we performed recordings using ACSF containing TTX and amino acid receptor antagonists. In the presence of TTX and amino acid receptor antagonists, the RMP (male, −68.9 ± 1.9 mV; female, −69.0 ± 1.6 mV; *p* = 0.9) and IR (male, 1.5 ± 0.2 GΩ, female, 1.2 ± 0.2 GΩ; *p* = 0.4) of ARH kisspeptin neurons did not differ between sexes. Therefore, male and female data were plotted together. Interestingly, IGF‐1 did not affect the activity of ARH kisspeptin neurons in the presence of TTX and amino acid receptor antagonists (Figure 5A–D), suggesting that IGF‐1 modulates the ARH kisspeptin neuronal activity indirectly (male, *n* = 10 cells out of 6 animals; female, *n* = 8 cells out of 5 animals).

## 4 DISCUSSION

Numerous studies have investigated the influence of various hormones and neurotransmitters on the activity of ARH kisspeptin neurons (presumptive KNDy cells).^30^, ^32^, ^41^, ^42^, ^43^, ^44^, ^46^, ^47^, ^56^, ^61^, ^67^, ^68^ While significant progress has been made in understanding the physiology of kisspeptin neurons, whether IGF‐1 modulates the HPG axis through the modulation of KNDy cells was not previously determined. We showed that kisspeptin neurons express IGF1R and that central administration of IGF‐1 induced LH secretion in male mice. While central administration of IGF‐1 did not significantly increase *Kiss1* mRNA levels in adult mice, acute IGF‐1 administration to brain slices was sufficient to elevate [Ca^2+^]i in ARH kisspeptin neurons and induce depolarization of their RMP. Nonetheless, at least part of IGF‐1's effects on ARH kisspeptin cell activity may be indirect, as TTX and amino acid receptor antagonists prevented its influences on KNDy neuron activity.

Previous studies have proposed a dual mechanism through which IGF‐1 may modulate the HPG axis. In prepubertal female rats, central or systemic IGF‐1 administration upregulates *Kiss1* mRNA expression in the AVPV but not in the ARH, which was proposed to influence puberty timing. Additionally, IGF‐1 stimulates LH release.^69^ A preprint reported similar findings in mice, showing that selective, inducible ablation of *Igf1r* in kisspeptin cells delays puberty in both female and male animals. Interestingly, serum LH levels remained unchanged in females, whereas males exhibited reduced LH secretion.^70^ Consistent with these observations, studies in monkeys showed that IGF‐1 influenced puberty maturation by advancing the age of first ovulation.^18^ In contrast, in adult rats, sheep, and monkeys, IGF‐1 modulates the HPG axis by stimulating LH release despite no evident effect on hypothalamic *Kiss1* mRNA levels, as observed in the current study.^16^, ^18^, ^71^ Our research contributes to the understanding that hypothalamic kisspeptin neurons express *igf1r*. This suggests that the effect of IGF‐1 on LH secretion, as demonstrated previously,^12^, ^16^, ^17^, ^18^ may rely on its interaction with kisspeptin neurons. Additionally, age may influence this effect, as the IGF‐1 icv administration did not modulate *Kiss1* mRNA expression in adult animals. Given that kisspeptin neurons express *igf1r*, this underscores the broader implications of our research for understanding the hormonal regulation of reproduction.

IGF‐1 administration significantly increased [Ca^2+^]i in a notable percentage of KNDy neurons recorded from brain slices. Changes in [Ca^2+^]i have been shown to correlate with changes in action potential firing, as IGF‐1 administration depolarized KNDy neurons. The IGF‐1‐induced depolarization of KNDy neurons was followed by a slight, albeit not statistically significant, increase in the *fAPs* when recordings were made in brain slices of female mice. We and others have previously demonstrated that most ARH kisspeptin cells are quiescent at rest.^64^, ^72^ Notably, in the present study, the proportion of quiescent cells before IGF‐1 administration was lower than reported in earlier studies.^64^, ^72^ This discrepancy may be attributed to the internal solution used here, which was employed based on the native intracellular chloride concentration in KNDy neurons, as determined using gramicidin‐perforated patch recordings.^73^ Nonetheless, the number of cells exhibiting APs and responsive to IGF‐1 in the current study is relatively low, which is a limitation of the study. This outcome, however, is directly related to the intrinsic biophysical properties of KNDy cells.^64^, ^72^ Importantly, the proportion of IGF‐1‐responsive cells identified through [Ca^2+^]i analysis was higher than the data obtained with the whole‐cell patch‐clamp technique. Fura‐2 can capture dynamic changes in intracellular calcium transients that reflect more than just cell depolarization, as observed in patch‐clamp experiments. Multiple sources and mechanisms can contribute to the observed results obtained with the Fura‐2. The mobilization of intracellular Ca^2+^ from internal stores, without requiring membrane depolarization, extracellular Ca^2+^ influx, or excitatory synaptic input leading to subthreshold postsynaptic potentials causing Ca^2+^ influx, are examples, among others. Therefore, we cannot discard the hypothesis that the observed upregulation of [Ca^2+^]i on KNDy cells can also be attributed to Ca^2+^ entry via voltage‐gated Ca^2+^ channels or Ca^2+^ release from internal stores. The differences observed when comparing IGF‐1's effects on [Ca^2+^]i levels and RMP changes reflect the inherent variability between the two technical approaches and cannot be directly compared. Importantly, a dose–response analysis was not performed. Therefore, we cannot exclude the possibility that the observed effects may vary with IGF‐1 concentration.

An indirect mechanism of action for IGF‐1 is suggested, as its effects on KNDy cells' RMP were blocked by TTX and amino acid receptor antagonists. Moreover, AVPV kisspeptin neurons were not responsive to IGF‐1. Given that the electrophysiological recordings were performed in brain slices, which results in the loss of many synaptic connections, a few considerations must be made. The observed IGF‐1 effect on KNDy neurons may stem from a specific neuronal population within the same brain slice that, when activated through IGF1R, transmits information to KNDy neurons. In this context, IGF‐1 may act via NPY/AgRP or POMC neurons, both of which are located in the ARH. Since NPY/AgRP and POMC neurons express the IGF1R,^74^, ^75^ these cells could represent a route by which IGF‐1 modulates KNDy neurons and the HPG axis. Given that NPY/AgRP exerts inhibitory effects on KNDy neurons, any modulation via this pathway would require a decrease in NPY/AgRP activity or peptide release to account for the observed depolarization of KNDy neurons to occur.^30^, ^33^ In contrast, kisspeptin neurons coexpress melanocortin receptors type 3 (MC3R) and type 4 (MC4R), which bind α‐melanocyte‐stimulating hormone (αMSH), a peptide derived from POMC neurons.^37^, ^76^ Blocking the central αMSH pathway suppresses ARH *Kiss1* mRNA expression in prepubertal rats, while additional evidence shows that the MC4R agonists stimulate KNDy cell activity.^77^ Indeed, the selective, inducible ablation of *Mc4r* from kisspeptin cells results in impaired reproductive function, whereas re‐insertion of *Mc4r* into kisspeptin cells of MC4r null mice restores estrous cyclicity and LH pulsatility.^77^ Furthermore, mice that overexpressed central IGF‐1 exhibit downregulation of *Pomc* mRNA levels in the hypothalamus, among other effects.^78^ Together, these findings suggest that POMC neurons are potential upstream modulators of the IGF‐1 effects on KNDy cells via melanocortin‐dependent mechanisms.

GABA‐expressing neurons may also contribute to the observed indirect effects since GABA can depolarize KNDy neurons.^73^ Additionally, considering that IGF‐1 can act not only through IGF1R but also on the estrogen receptor (ER) via a ligand‐independent signaling pathway or with less affinity to the insulin receptor, multiple neural populations within the same brain slice, which have been shown to coexpress one of the mentioned receptors, could further contribute to the observed result.^1^, ^79^ Of note, KNDy neurons express the insulin receptor, and insulin receptor ablation in kisspeptin‐expressing cells alters puberty timing without impairing fertility.^45^ Therefore, the observed IGF‐1 effects on KNDy neurons seem independent of insulin receptor activation, at least on kisspeptin‐expressing cells, since TTX and amino acid receptor antagonists prevented the effects on RMP.

Pieces of evidence further place glial cells as potential candidates to mediate IGF‐1 effects on KNDy neuron activity. Glial cells in the preoptic area and mediobasal hypothalamus express the IGF1R.^80^ In addition, IGF‐1 immunoreactivity levels increase in ARH glial cells during puberty, in the afternoon of proestrus, and the morning of estrus.^81^ Furthermore, central administration of IGF‐1 upregulates the transcription factors Oct2a and Oct2c, which are involved in glial‐neuronal regulatory processes that contribute to GnRH secretion in prepubertal female rats.^82^ Conversely, kisspeptin modulation of astrocyte activity is essential for maintaining pulsatile LH secretion.^83^ The definition of which neural population(s) modulate(s) the effects of IGF‐1 on KNDy neurons, or can be recruited to induce a fast LH secretion, as reported,^12^, ^16^, ^17^, ^18^ needs further investigation. Importantly, the current experiments were carried out with gonad‐intact animals, so variations in sex hormone levels may represent a confounding factor in the responses observed and should be considered in our conclusions. Therefore, experiments often use castrated animals treated with physiological levels of sex steroids, which provides better control over *Kiss1* gene expression and reproductive functions. However, it is also essential to perform experiments with gonad‐intact animals, as they represent a natural physiological state that is often challenging to fully replicate with hormone replacement therapy.

In conclusion, the precise mechanisms by which various physiological signals are integrated to modulate the HPG axis under unfavorable nutritional conditions remain incomplete. Here, we contributed to the field by demonstrating that the IGF‐1 effect on the KNDy neuron activity of adult mice is indirect, despite these cells expressing *igf1r*. Exploring IGF‐1's role in modulating KNDy neurons offers a new piece into the neuroendocrine networks involved in reproduction. These can have broader implications for conditions in which metabolism affects reproduction, such as undernutrition, obesity, and polycystic ovary syndrome. Adding to the literature, insights into how IGF‐1 modulates KNDy neuron activity can, in the long term, reveal potential targets for therapeutic interventions in reproductive disorders, particularly those linked to metabolic issues or the GH/IGF‐1 axis.

## CONFLICT OF INTEREST STATEMENT

The authors declare no conflict of interest.

## Data Availability

The data supporting this study's findings are available from the corresponding author upon reasonable request.
